# Digging deeper - A new data mining workflow for improved processing and interpretation of high resolution GC-Q-TOF MS data in archaeological research

**DOI:** 10.1038/s41598-019-57154-8

**Published:** 2020-01-21

**Authors:** Ansgar Korf, Simon Hammann, Robin Schmid, Matti Froning, Heiko Hayen, Lucy J. E. Cramp

**Affiliations:** 1 Institute of Inorganic and Analytical Chemistry, University of Münster, Corrensstraße 30, 48149 Münster, Germany; 2 Department of Anthropology and Archaeology, University of Bristol, 43 Woodland Road, Bristol, BS81UU UK; 3 Present Address: Department of Chemistry and Pharmacy, Friedrich-Alexander University Erlangen-Nürnberg, Nikolaus-Fiebiger-Straße 10, 91058 Erlangen, Germany

**Keywords:** Data mining, Analytical chemistry, Mass spectrometry, Archaeology

## Abstract

Gas chromatography-mass spectrometry profiling is the most established method for the analysis of organic residues, particularly lipids, from archaeological contexts. This technique allows the decryption of hidden chemical information associated with archaeological artefacts, such as ceramic pottery fragments. The molecular and isotopic compositions of such residues can be used to reconstruct past resource use, and hence address major questions relating to patterns of subsistence, diet and ritual practices in the past. A targeted data analysis approach, based on previous findings reported in the literature is common but greatly depends on the investigator’s prior knowledge of specific compound classes and their mass spectrometric behaviour, and poses the risk of missing unknown, potentially diagnostic compounds. Organic residues from post-prehistoric archaeological samples often lead to highly complex chromatograms, which makes manual chromatogram inspection very tedious and time consuming, especially for large datasets. This poses a significant limitation regarding the scale and interpretative scopes of such projects. Therefore, we have developed a non-targeted data mining workflow to extract a higher number of known and unknown compounds from the raw data to reduce investigator’s bias and to vastly accelerate overall analysis time. The workflow covers all steps from raw data handling, feature selection, and compound identification up to statistical interpretation.

## Introduction

Fragments from unglazed ceramic cooking and storage pots (pot sherds), are one of the most common artefact-types recovered at archaeological excavations^1^. Besides the chronological and other information originating from visible features of these sherds, they also contain hidden chemical information that reflects their use history. Absorbed in the inorganic matrix and protected from microbial degradation and water leaching, residues of lipids (and other food constituents) can be preserved over millennia^1,2^. These accumulated lipid residues are an important source of information and allow reconstruction of the original vessel contents and thus the dietary, ritual and food procurement practices of past populations^3–5^.

To achieve this, lipids are extracted from powdered pottery samples and the molecular and isotopic composition is determined. Critically, only a small fraction of the originally absorbed lipids is actually preserved and can be recovered. Frequently, they have undergone structural changes. For instance, unsaturated fatty acids, although abundant in most food lipids, are only rarely recovered due to their higher susceptibility to oxidative degradation^6^. Similarly, hydrolytic changes occur, leading to a decrease of ester lipids (such as triacylglycerols or wax esters) and a dominance of their hydrolysis products, most prominently saturated fatty acids^7,8^. Thus, palmitic acid (16:0) and stearic acid (18:0) are the most frequently recovered lipids from archaeological pottery matrices. While these are not diagnostic by themselves, different biosynthetic pathways and carbon routings between non-ruminant (e.g. pig) and ruminant animals (e.g. cattle), as well as between adipose tissue and mammary glands, lead to different carbon isotopic compositions^9^. Using compound-specific stable isotope techniques, such as gas chromatography-combustion-isotope ratio mass spectrometry (GC-C-IRMS), differences in δ^13^C values can be exploited to distinguish lipids in pots originating from the processing of porcine and cattle adipose lipids or dairy products^9^. In addition, some easily degradable compounds such as polyunsaturated fatty acids can form highly diagnostic and stable transformation products. In reference experiments it has been shown how heating of long-chain polyunsaturated and monounsaturated fatty acids (as commonly encountered in aquatic lipids) can form a series of ω-(*o*-alkylphenyl)-alkanoic acids (APAAs) and vicinal dihydroxy fatty acids^10–12^. While the original unsaturated fatty acids are almost never recovered, their degradation products are routinely used as proxies to infer the original presence of these lipids^5,13,14^.

**Figure 1:**
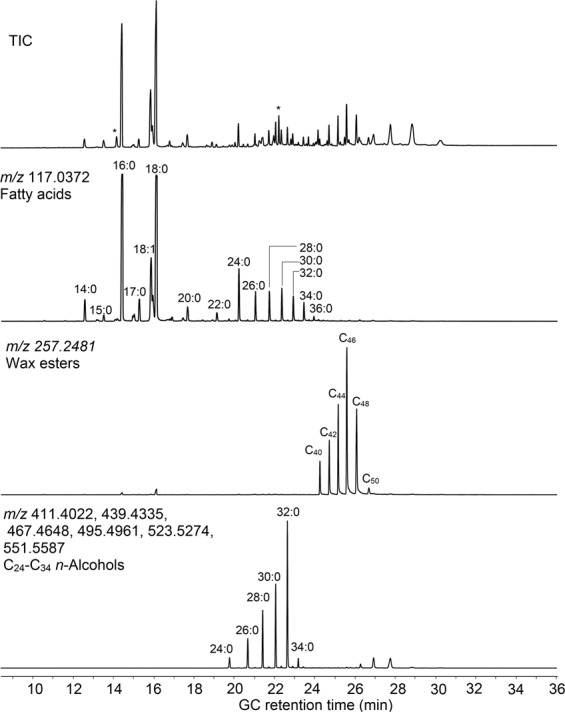
GC-Q-TOF MS chromatogram of a trimethylsilylated lipid extract from an archaeological sample displaying the total ion chromatogram (TIC, top) and the extracted ion chromatogram (EIC) for *m/z* 117.0372 (second panel), which shows the elution of trimethlysilylated fatty acids from C_14_–C_36_. The third panel (EIC of *m/z* 257.2481) and the fourth panel (sum of EIC *m/z* 411.4022, 439.4335, 467.4648, 495.4961, 523.5274 and 5511.5587) show the elution of wax esters and C_24_-C_34_ alcohols (trimethylsilylated), respectively. Peaks marked with an asterisk are internal standards.

Over its lifetime, a cooking pot can be used for several thousand individual cooking events, and it is most likely that ingredients were mixed or the same pot used sequentially for different commodities. The lipid pattern therefore reflects an accumulation of the lifetime usage of the pot, which can result in very complex lipid patterns. In addition, use-related changes as well as post-depositional degradation increases the complexity of the lipid patterns even further^1,2^. Consequently, lipid extracts from archaeological samples often contain several hundred individual compounds, which makes the analysis and interpretation very challenging (Fig. 1). The high separation power of gas chromatography (GC) can be effectively used to separate as many compounds as possible, and single quadrupole GC-MS has been used extensively to confirm peak identity^3,15,16^. However, the low spectral resolution of these instruments limits their use for identification of unknown compounds. Moreover, manual data interpretation is very common and especially in larger projects, where thousands of samples are analysed, this can be very tedious and time-consuming and minor diagnostic compounds are likely missed. This has the effect of either placing constraints upon the scale and scope of projects undertaken, or means that the full diagnostic potential of archaeological residues is often not being realised.

Recently, we have used GC coupled to a high-resolution quadrupole-time-of-flight mass spectrometer (GC-Q-TOF MS) for the targeted analysis of cereal biomarkers in archaeological samples and for non-targeted lipid profiling of modern cereal lipids^17,18^. We have now transferred and optimised our non-targeted lipid analysis workflow for archaeological samples and want to use this to address common limitations of the current state of the art in archaeological lipid research. We focus on advancements in automated data processing workflows, the creation and usage of open libraries for spectral matching, and data interpretation. This now offers the potential to enhance the interpretative value achievable through analysis of ancient organic residues.

The arrival of open-source LC-MS data mining software solutions, such as MZmine^19^ and XCMS^20^ in the mid 2000s has opened up new possibilities for rapid data processing. Originally designed for metabolomics, these data mining software packages were used for various fields of study. In particular, MZmine, now in its second generation^21^, stands out due to its modular design which allows straightforward software extension. Therefore, we have added automated spectra matching to MZmine 2, which was the missing piece required for high-throughput GC-MS data analysis workflows. In addition, we have collected spectra from available standard compounds, well-characterised archaeological, and modern (cereal) lipid samples in order to build a reference library for archaeologically relevant compounds. The library will be provided in various file formats to facilitate compatibility with MZmine 2 and other open-source and proprietary software solutions. The developed workflow will be exemplified on a dataset consisting of lipids from 40 ceramic samples from the site of Vindolanda (Northumberland, UK), a Romano-British defence fort south of Hadrian’s Wall.

## Experimental Section

### Chemicals

Chloroform, methanol, dichloromethane and *n*-hexane (all HPLC grade) were from Rathburn Chemicals (Walkerburn, UK), while tetratriacontane (>98%), pyridine, methyl hexadecanoate, methyl heptadecanoate, methyl eicosanoate, methyl docosanoate, trimyristate, tripalmitate, tristearate (all >99%), and the derivatisation agent consisting of *N*,*O*-bis(trimethylsilyl) trifluoroacetamide/trimethylchlorosilane (BSTFA/TMCS) 99:1 (*v/v*) were supplied by Sigma-Aldrich (Munich, Germany).

### Samples

Archaeological sherds were from the site of Vindolanda (Northumberland, UK), a Romano-British auxiliary defence fort south of Hadrian’s Wall. A total of 40 recently-excavated sherds from this site were selected in this study. The sherds date from the same phase of occupation (AD 105–120) and derive from the military context within the fort (n = 33), and the supposedly-local, civilian settlement that emerged outside the walls of the fort (n = 7).

### Sample preparation and lipid extraction

The sherds were cleaned using a modelling drill and crushed to a fine powder using a mortar and pestle. After adding 40 µg of tetratriacontane (C_34_ alkane) as internal standard, approximately 2 g of the powder were extracted under sonication using 2 × 10 mL chloroform/methanol 2:1 (*v/v*). After centrifugation, the supernatant was transferred into a glass vial and the solvent was removed under a gentle stream of nitrogen. The residue was then re-dissolved in 2 mL chloroform/methanol 2:1 (*v/v*). An aliquot of 0.5 mL was applied on a small glass column (1 cm i.d.) filled with 0.5 g activated silica (conditioned with 5 mL chloroform/methanol 2:1 (*v/v*)). Lipids were eluted with 5 mL chloroform/methanol 2:1 (*v/v*). The solvent was transferred into a glass vial and blown to dryness. To this residue, 25 µL dry pyridine and 50 µL of the silylating agent (BSTFA/TMCS 99:1, *v/v*) were added and heated at 70 °C for 1 h. The silylating agent was then removed under a stream of nitrogen, the residue was re-dissolved in 0.5 mL *n*-hexane and, after the addition of  2.5 µg of the second internal standard methyl heptadeconate, used for GC-flame ionization detector (FID) and GC-Q-TOF MS analysis.

### Reference library building

A reference library was built from available standard compounds, well-characterised archaeological, and modern (cereal) lipid samples. Where possible, deconvoluted and background-subtracted spectra were used. Spectra were only manipulated to remove clearly identifiable background or noise signals. Structures were assigned to best knowledge and probability, but it needs to be noted that in electron ionisation (EI) neither the position nor orientation of double bonds in fatty acids nor the *sn*1/*sn*2 distribution of fatty acids in triacylglycerols can be reliably assigned (See “Limitations” below). The reference library can be accessed at https://gc-hrms-spectra.github.io/.

### Analysis of trimethylsilylated lipid extracts by GC-FID and GC-Q-TOF MS

Extracted lipids were analysed after trimethylsilylation by GC-FID as described before in detail^18^. Lipids were also analysed by GC-Q-TOF MS as described before^17^. In short, trimethylsilylated aliquots of the lipid extracts were analysed using a 7890/7200B GC-Q-TOF MS (Agilent, Santa Clara, CA, USA) and a 15 m, 0.25 mm i.d., 0.1 µm film thickness ZB-5HT Inferno column (Phenomenex, Torrence, CA, USA). Data (profile and centroid) was recorded in the Extended Dynamic Range mode with 5 scans/s. The carrier gas flow rate, temperature program, and mass spectrometry conditions were identical to those described before. A standard consisting of methyl hexadecanoate, methyl eicosanoate, methyl docosanoate, tetratriacontane, trimyristate, tripalmitate, and tristearate was analysed with every sample batch for quality control and to assess inter- and intra-batch variation of chromatographic and mass spectrometric performance.

## Results and Discussion

### Analysis of archaeological lipids using GC-Q-TOF MS

Lipids could be extracted from all samples in appreciable quantities and lipid contents varied between 24 and 1383 µg/g ceramics (determined by GC-FID). Using a 15 m column with a non-polar stationary phase also allowed the elution of intact ester lipids, such as triacylglycerols (C_42_–C_54_) and wax esters. However, many samples featured a very complex lipid pattern with ≫100 partly resolved peaks, which made compound identification based on GC retention times alone difficult and not advisable. GC-MS not only allowed confirmation of peak identities through the respective mass spectra, but also deconvolution of co-eluting peaks. Importantly, the higher sensitivity and selectivity through the accurate mass capabilities of the instrument allowed detection of further minor compounds previously not detected. By extracting the ion traces of *m/z* 117.0372 and 257.2481 for example, the distribution of minor very long chain fatty acids and esters of palmitic acid with long-chain alcohols, respectively, could be investigated. In the extracted residue shown in Fig. 1, the distribution of fatty acids, wax esters, and alcohols (together with other characteristic compounds) indicated the presence of beeswax in this particular pot.

While this approach is very powerful to be used in a more targeted manner, it depends on the investigator’s prior knowledge of specific compound classes and their mass spectrometric behaviour to select appropriate ion traces, and unknown compounds will often be missed completely. This is important since the diagnostic potential of minor compounds over more ubiquitous major compounds is becoming increasingly recognised^12,18,22^. Furthermore, this approach can be very tedious and time consuming for a high number of samples and compounds (or compound classes) that need to be investigated. Non-targeted data mining workflows can help to extract a higher number of known and unknown compounds from the raw data and therefore not only reduce investigator’s bias but also vastly accelerate overall analysis time. In addition, these unknown compounds can potentially contain valuable information in archaeological contexts, which can be made accessible through dedicated data processing and interpretation procedures. Therefore, a new GC-MS data mining workflow was developed, which added new algorithms and functionalities to established tools.

### Optimization of a LC-MS metabolomics data mining workflow for GC-MS data

The dataset was converted to the open format mzML^23^, using the MSConvert software of the ProteoWizard toolkit^24^. The conversion is necessary to ensure MZmine compatibility. The converted dataset can be processed with various peak picking software tools, such as MZmine^19,21^, XCMS^20^, or OpenMS^25^. Due to its open-source modular framework, MZmine 2 has seen multiple extensions implemented by various different laboratories in the past years, which include feature detection algorithms^26–28^, molecular networking^29,30^, visualization techniques^31,32^, as well as compound identification algorithms^33,34^, making the overall toolbox almost ready for GC-TOF MS data mining of complex archaeological sample sets, as recently shown by Decq *et al*.^35^. Since electron ionisation (EI) results in numerous fragments, which provide information about the molecular structure, spectra matching was the choice for compound identification. As MZmine has its roots in LC-MS profiling of metabolomics datasets, automatic spectral library matching was not yet supported. In addition, there was no high-resolution spectral GC-MS library specific enough for archaeological biomarkers. Thus, we created a spectral reference library and added spectra matching functions to MZmine 2. The created spectral library is available at https://gc-hrms-spectra.github.io/. Spectra matching support is available since MZmine 2.39, which was further improved and optimized for GC-MS in versions 2.40 and 2.41. In combination with the already existing export module for MetaboAnalyst, the processed and annotated feature lists can be statistically evaluated^36^.

**Figure 2:**
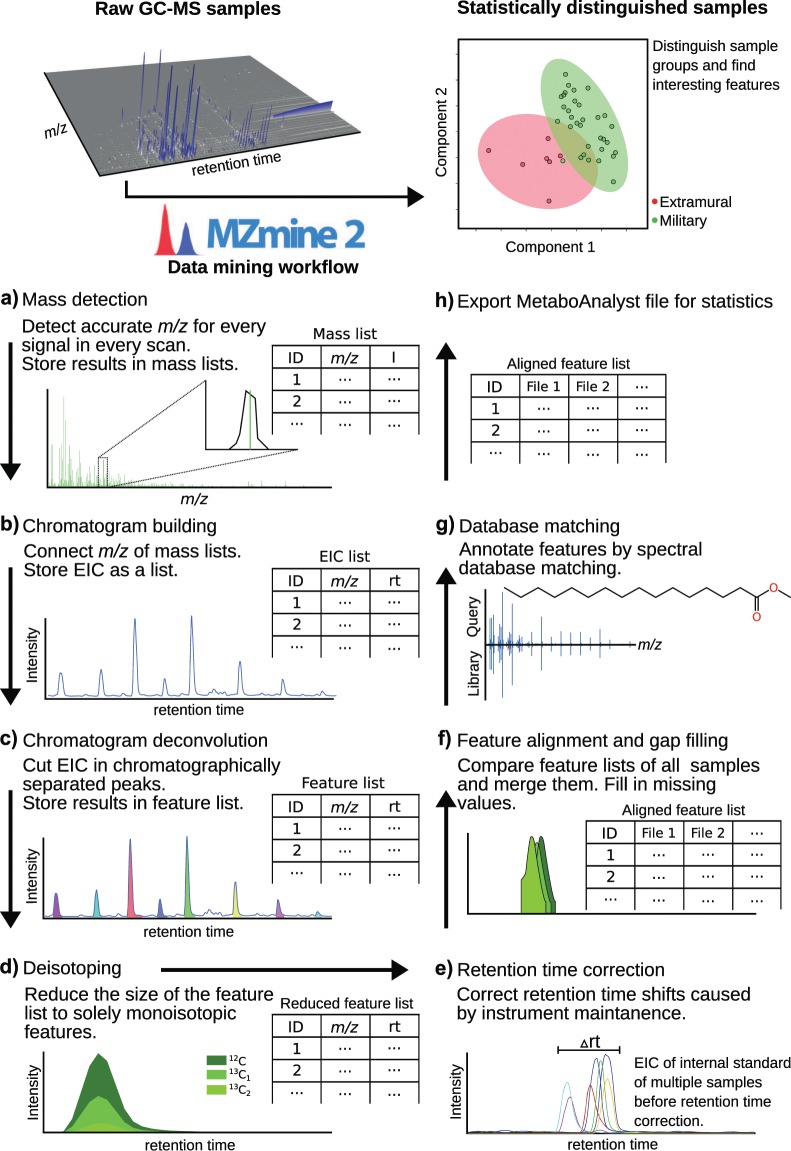
GC-MS data mining workflow. The workflow covers all steps from raw data handling in MZmine 2 to statistical interpretation in MetaboAnalyst.

Figure 2 displays the overall data mining workflow, covering all steps from raw data handling to statistical evaluation. First (Fig. 2a), each accurate *m/z* is determined for each signal in each scan above a user-set noise level. The resulting pairs of *m/z* and intensity are stored in so-called mass lists. In a second step (Fig. 2b), the mass lists of the individual scans are connected to EICs, which are stored in a list that can be examined by the user. Due to the nature of EI as a “hard” ionisation technique, numerous fragments are formed for all compounds, which can be very similar or identical for different lipids. For example, all trimethylsilylated fatty acids form a common fragment ion detected at *m/z* 117.0372 (C_4_H_9_O_2_Si^+^) through a cleavage between C-1 and C-2. Therefore, it is necessary to deconvolute EICs with multiple peaks into chromatographically separated features, as displayed in Fig. 2c. Due to the natural occurrence of isotopes, the same compounds are represented by several features with different isotopic compositions. Therefore, these features are grouped in the fourth step (Fig. 2d) and are represented by the feature with the monoisotopic composition. Another challenge with large GC-MS datasets are retention time shifts caused for example by instrument maintenance. By using internal standards, these shifts can be corrected automatically as depicted in Fig. 2e. This correction of the data heavily improves the results of the next steps, namely, feature alignment and gap filling. These algorithms merge all feature lists from all analyzed samples into a single data matrix (Fig. 2f). In addition, the raw data for each gap is checked again to ensure that a feature was not erroneously removed when processing the data. Even if this was not the case, at least the noise level is added to improve the statistical results. Next, the aligned feature list rows can be annotated using the newly implemented spectral library matching module (Fig. 2g).

**Figure 3:**
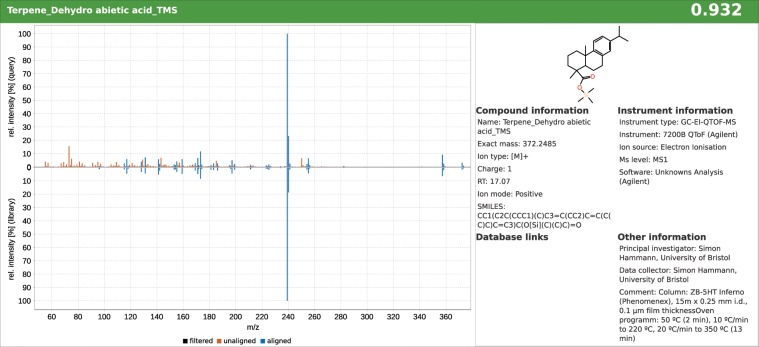
The MZmine 2 library matching result panel for dehydroabietic acid compiles a spectra mirror plot of an experimental scan (left, top) and a library scan (left, bottom), the structure and metadata of the library entry (right) and the spectral similarity (0.932) in the top right corner.

Figure 3 displays the library matching result panel of MZmine 2 for the terpenoid dehydroabietic acid (as trimethylsilyl derivative) in one of the samples. The match result consists of two main panels, a spectra mirror plot on the left, showing the experimental scan (top) and the matched library scan (bottom), and on the right a metadata panel, which depicts the structure of the identified molecule and various compound and method specific information. In the mirror plot, blue signals are matched with the library and orange signals are unmatched. In addition, a spectral similarity score is given in the upper right corner. The score is based on the composite similarity^37^ and ranges from 0 to 1, for completely dissimilar to identical, respectively. Hence, the similarity score of 0.932 depicted in Fig. 3 (top, right) denotes a high resemblance of the experimental and the library scan.

In a last step, the annotated aligned feature list can be exported using the provided export function for MetaboAnalyst (Fig. 2h). Subsequently, statistics can be easily performed using the free MetaboAnalyst 4.0 online platform^36^.

**Figure 4:**
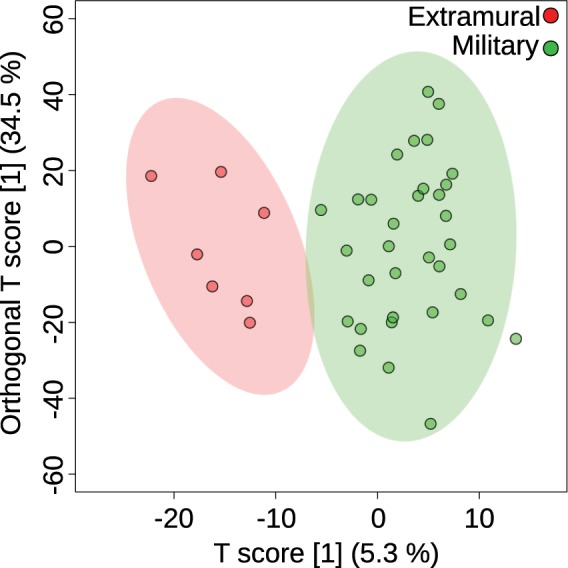
Scores plot of ortho PLS-DA showing the discrimination of extramural (red) and military (green) samples based on their GC-Q-TOF MS lipid profiles.

The described workflow was performed using the 40 samples from the Vindolanda dataset. Supervised multivariate statistics, such as Partial Least Squares - Discriminant Analysis (PLS-DA), ortho PLS-DA^38,39^ (only for two sample groups) or sparse PLS-DA (sPLS-DA)^40^ can be used to discriminate the two sample groups (military and extramural), as displayed in Fig. 2, top right using sPLS-DA and in Fig. 4 using ortho PLS-DA.

**Figure 5:**
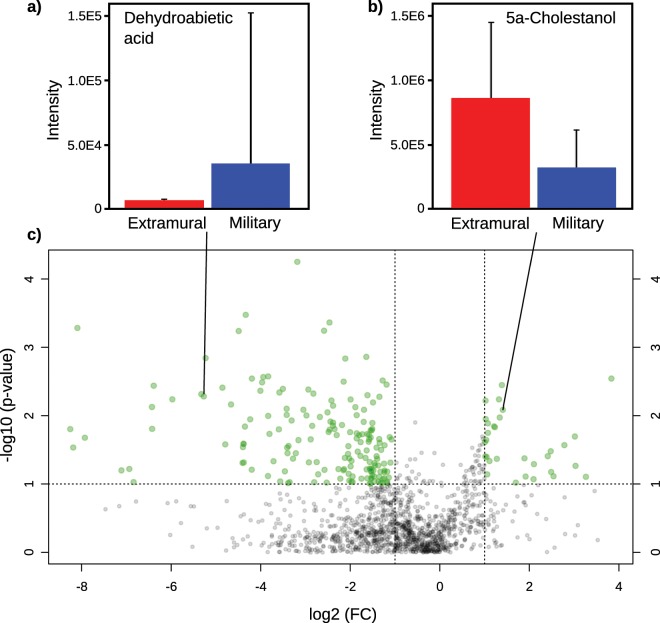
(**a**) Bar chart plot of dehydroabietic acid, which is more abundant in military samples (blue). (**b**) Bar chart plot of 5α-cholestanol, which is more abundant in extramural samples (red). (**c**) Volcano plot of the aligned feature list of 40 samples from the site of Roman Vindolanda.

The loadings plot of the respective scores plot can be investigated to identify significantly differing compounds across sample groups. In the case of two sample groups, other statistical methods, such as volcano plots can be used as a powerful tool to rapidly identify significant changes in a compound’s intensity across sample groups, as displayed in Fig. 5c. A volcano plot combines fold-changes (FC), displayed on the x-axis, and the significance (t-test) of these changes, depicted as -log10(p-value) on the y-axis, in a single scatter plot. In Fig. 5c compounds above user defined thresholds for FC (2.0) and p-value (0.1) are highlighted in green and the thresholds are marked as dotted lines. The volcano plot in Fig. 5c shows numerous significant compounds. Interesting compounds in the archaeological context were, for example, dehydroabietic acid (Fig. 5a), which was significantly more abundant in the military samples compared to the extramural samples.

Dehydroabietic acid, identified through the intensive fragment ion detected at *m/z* 239.1794 (C_18_H_23_^+^) and the [M-15]^+^ fragment ion detected at *m/z* 357.2250 (C_22_H_33_O_2_Si^+^), is a stable compound formed from terpenoic acids that are commonly found in conifer resins, including that of pine trees (*Pinaceae*). Its presence in archaeological ceramics is seen as a proxy for the presence of these resins, where they could have been used as sealing and waterproofing agents, as well as for flavouring, ritual balsams and even exploited for their antimicrobial properties. Due to the absence or low abundance of related compounds, such as retene and dehydroabietic acid methyl ester, the material used was more likely a resin and not a heated pine pitch. The absence of 7-oxo-dehydro-abietic acid can be explained through the anoxic conditions at the site^41–43^. Use of coniferous resins is known to have been widespread in the Roman world, and its presence has been determined in pottery absorbed residues^44^ and amphorae internal coatings^45^, as well as from mortuary ‘grave dust’ from Roman Britain^46^.

In contrast, 5α-cholestanol, identified through GC retention time, an intensive fragment ion at *m/z* 215.1794 (C_16_H_23_^+^) and the [M-15]^+^ ion at *m/z* 445.3866 (C_29_H_53_OSi^+^), was significantly more abundant in the extramural samples (Fig. 5b). 5α-Cholestanol is the biohydrogenation product of the principal animal sterol, cholesterol, and due to its fully saturated ring system it is less susceptible to oxidative degradation than its parent molecule, which favours its preservation^47^. The higher levels of this molecule in the extramural samples could be either due to higher initial levels of cholesterol, or better preservation. However, it was not found that cholesterol itself was more abundant in extramural samples. Therefore, a likely explanation is that this is evidence of different post-depositional conditions, e.g., stronger reducing than oxidising conditions, in the extramural settlement compared to the fort itself. This demonstrates the value of lipids as molecular fossils in archaeological research.

### Application of the new workflow and limitations

This workflow is designed to guide towards analytically important and significant features, and can significantly speed up the processing of large sample sets. However, certain limitations of this workflow need to be considered. GC-EI-MS has inherent caveats when it comes to structural identification of lipids and good library matches can sometimes give a false sense of specificity. For example, virtually all monounsaturated C_18_ fatty acids will show the same mass spectrum, disregard of double bond position or orientation (*cis*/*trans*). Similarly, the spectra of *n-*15:0 and *iso*/*anteiso*-15:0 (13-methyl- and 12-methyl-14:0, respectively) exhibit very little spectral difference. Furthermore, triacylglycerols show molecular ions only at very low intensity and the main fragments stem from the elimination of one or two acyl chains. However, these fragments are often identical, for example for a C_54_ TAG after elimination of a C_18_ fatty acid and for a C_52_ TAG after elimination of a C_16_ fatty acid and this makes reliable library matching very difficult. In addition, different instruments or instrument settings can have a big impact on ratios of fragment ions. Therefore, results from this workflow still need some manual checking for plausibility and should never just be accepted with blind trust. In particular, GC elution orders need to be considered, and the use of a standard mix for retention time referencing is also highly encouraged. In this way, this workflow should be considered a starting point and used to guide the researcher towards interesting compounds, which should be further investigated and (manually) verified. Furthermore, the data mining workflow presented in this work considers every generated ion as an independent feature. The advantage of this is that the raw data can be mapped accurately. Smirnov *et al*. have also developed and implemented algorithms in MZmine 2 to construct deconvoluted GC-MS spectra^28,48^. These algorithms can be subsequently applied or potentially implemented in the workflow to further improve non-targeted compound identification in archaeology.

## Conclusion

The developed workflow has enabled the rapid identification of significant compounds in archaeological samples acquired by GC-Q-TOF MS. The workflow was exemplified on a dataset of 40 sherds from the site of Vindolanda (Northumberland, UK), a Romano-British defence fort south of Hadrian’s Wall. The contemporaneous pots were excavated from either a military context within the fort (n = 33) or the nearby *vicus*, likely inhabited by the local, non-military population (n = 7). A discrimination of these two sample groups was possible based on non-targeted GC-Q-TOF MS lipid profiles using supervised multivariate statistics on the resulting data matrix of the data mining workflow. This revealed significantly higher levels of dehydroabietic acid in the military samples, which shows a wider presence of conifer resins in this group, possibly from storage or preparation in resinous vessels. In contrast, the higher levels of 5α-cholestanol in the extramural samples hints towards slightly different preservation (or soil conditions) between these sample groups. The workflow therefore is very useful to guide the researcher towards the significant features among the dozens or hundreds of undiagnostic compounds. This, together with the newly created open spectra database, considerably improves interpretation of the complex lipid distribution frequently encountered in archaeological research and allows extraction of considerably more information and improved interpretations of the results.

## Data Availability

The GC-HRMS library used in this study is freely available online (https://gc-hrms-spectra.github.io/), and the spectra matching module is now integrated within MZmine 2 (since version 2.39). The raw GC-MS data used in this study will be available at 10.5523/bris.26hh9g6ktji7z2r5gxb2wqvjfq. Data is embargoed until 1 July 2021.
